# Curcumin Ameliorates Particulate Matter-Induced Pulmonary Injury through Bimodal Regulation of Macrophage Inflammation via NF-κB and Nrf2

**DOI:** 10.3390/ijms24031858

**Published:** 2023-01-17

**Authors:** Min Kook Lee, Hyo Dam Kim, Suk Hee Lee, Jin Hyup Lee

**Affiliations:** 1 Department of Food and Biotechnology, Korea University, Sejong 30019, Republic of Korea; 2 BK21 FOUR Research Group for Omics-Based Bio-Health in Food Industry, Korea University, Sejong 30019, Republic of Korea; 3 Biological Clock-Based Anti-Aging Convergence RLRC, Korea University, Sejong 30019, Republic of Korea

**Keywords:** curcumin, particulate matter, macrophage, pulmonary inflammation, oxidative stress

## Abstract

The direct effects of particulate matter (PM) on lung injury and its specific molecular mechanisms are unclear. However, experimental evidence has shown that oxidative stress-mediated inflammation in macrophages is the main pathological outcome of PM exposure. Curcumin has been reported to protect organs against the disturbance of homeostasis caused by various toxic agents through anti-inflammatory and antioxidative effects. However, the protective action of curcumin against PM-induced pulmonary inflammation and the underlying mechanism have not been thoroughly investigated. In this study, we established a PM-induced pulmonary inflammation mouse model using the intratracheal instillation method to investigate the protective ability of curcumin against PM-induced pulmonary inflammation. Compared to the mice treated with PM only, the curcumin-treated mice showed alleviated alveolar damage, decreased immune cell infiltration, and reduced proinflammatory cytokine production in both lung tissue and BALF. To evaluate the underlying mechanism, the mouse macrophage cell line RAW264.7 was used. Pretreatment with curcumin prevented the production of PM-induced proinflammatory cytokines by deactivating NF-κB through the suppression of MAPK signaling pathways. Furthermore, curcumin appears to attenuate PM-induced oxidative stress through the activation of Nrf2 and downstream antioxidant signaling. Our findings demonstrate that curcumin protects against PM-induced lung injury by suppressing oxidative stress and inflammatory activation in macrophages.

## 1. Introduction

Air pollution is a global environmental health risk factor [1]. The Lancet Commission on pollution and health estimated that approximately 9 million excess deaths worldwide are attributable to degraded environmental conditions, mainly ambient air pollution [2]. Airborne particulate matter (PM), a dominant air pollutant, is the fifth leading risk factor, causing 4.2 million deaths annually according to a Global Burden of Disease report [3,4]. Epidemiological studies have demonstrated that exposure to PM is associated with morbidity, mortality, and hospitalization due to a wide range of detrimental effects on the cardiovascular and respiratory systems [5,6,7].

Although the underlying mechanisms of PM-induced lung damage are not fully understood, oxidative stress and inflammation are pathophysiological factors of PM exposure. PM generates oxygen free radicals from the particle surface and disrupts cellular homeostasis, inducing the formation of reactive oxygen species (ROS) from mitochondria and the depletion of antioxidant defenses, and eventually inducing oxidative stress [8,9,10]. Inflammation interacts with oxidative stress for many disease processes, but it is also a key pathway in the biological effects of inhaled PM [11]. The exposure of macrophages, the first-line and predominant defender of the immune reaction in organs, including lung tissue, to PM causes intracellular ROS, resulting in a proinflammatory response through the activation of nuclear factor kappa-light-chain-enhancer of activated B cells (NF-κB) [12,13,14]. Consequently, PM-activated macrophages produce proinflammatory cytokines, such as interleukin-1β, IL-6, and tumor necrosis factor-α (TNF-α), and release nitric oxide (NO), a signaling molecule that regulates inflammation, thereby promoting lung injury [15,16].

It is well known that antioxidants can effectively neutralize the inflammatory response in stimulated macrophages and that natural food-derived antioxidants are the most useful. Indeed, various natural compounds, such as polyphenols, flavonoids, and anthocyanins, suppress inflammation by regulating oxidative stress [17,18,19,20]. Curcumin is a well-known plant-derived polyphenol with a wide range of activities, including antibacterial, anti-inflammatory, antitumor, and antioxidant activities [21,22,23]. In recent years, many studies have indicated that curcumin attenuates the production of inflammatory molecules by regulating oxidative stress in in vitro models [24,25,26]. Furthermore, these antioxidant and anti-inflammatory properties of curcumin appeared to be important targets to improve PM-induced diseases. For example, pretreatment of curcumin in human nasal fibroblast reduces ROS production caused by PM in a dose-dependent manner, suggesting that curcumin may be useful in the treatment of PM-induced nasal diseases [27]. Curcumin also protects BEAS-2B cells against PM-induced oxidative damage and apoptotic cell death by promoting the activation of NRF2 pathways [28].

However, the effects of curcumin on PM-induced lung injury in the context of a detailed mechanism in macrophage cells, the first responder to PM exposure and the predominant producer of inflammatory cytokines, have not been sufficiently investigated. In the present study, we investigated the protective effects of curcumin against PM-induced pulmonary injury and the anti-oxidative-stress and anti-inflammatory effects of curcumin on macrophages, both in vitro and in vivo. An improved understanding of the multiple functions of curcumin in PM-induced pulmonary inflammation may facilitate the prevention and treatment of PM-induced lung diseases.

## 2. Results

### 2.1. Curcumin Alleviated Histological Changes and Immune Cell Infiltration in PM-Treated Mouse Lung Tissue and Bronchoalveolar Lavage Fluid (BALF)

**Figure 1:**
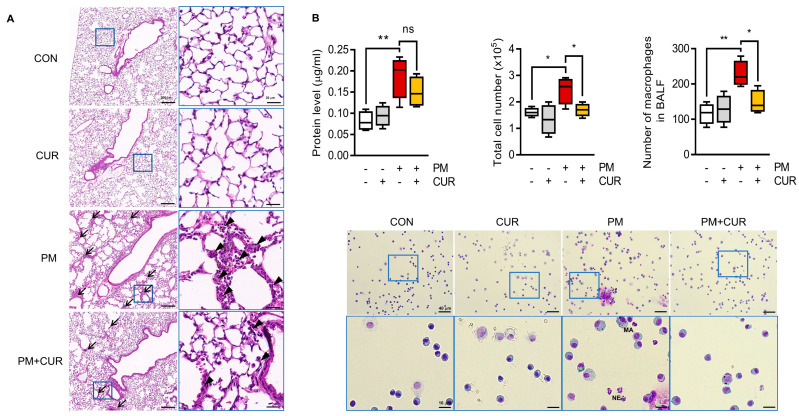
Curcumin alleviated histological change and immune cell infiltration in PM-treated mice lung tissue and BALF. (**A**) Effect of curcumin on pathohistological changes after bronchial administration of PM or saline. Mice were euthanized and lung tissues were stained with H&E to observe inflammatory aspects. Images show low- (**left**; scale bars, 200 μm) and high-power magnification (**right**; scale bars, 30 μm). Inflammatory foci marked with arrow and infiltrated immune cells marked with arrowhead. (**B**) BALF were harvested for quantification of protein concentration and total cell number. The ratio of macrophages to total cells was determined after Giemsa staining. Images show 100× (upper; scale bars, 40 μm) and 400× magnification (lower; scale bars, 10 μm). ** *p* < 0.01, * *p* < 0.05, and ns (not significant) indicates *p* ≥ 0.05 vs. PM-treated group. CON, control group; CUR, curcumin; PM, particulate matter (n = 6 per group).

Mice were intratracheally exposed to PM, with or without oral curcumin pretreatment. Hematoxylin and eosin (H&E)-stained lung sections were microscopically observed to clarify the histopathological alterations in each group. The control (CON) and curcumin only (CUR) groups showed a normal alveolar morphology, while the PM-exposed group (PM) exhibited obvious inflammatory lesions, indicated by the thickened alveolar wall with decreased airspace area, and the accumulation of multiple inflammatory cells in the alveolar space (Figure 1A). In contrast, the group of mice that received curcumin orally prior to PM exposure (PM+CUR) showed attenuated PM-induced inflammatory responses. In addition, the protein concentration and total cell number in BALF, primary indicators of pulmonary inflammation, were measured, as the influx of increased numbers of various leukocytes characterizes lung inflammation. Figure 1B shows that the PM group possessed an increased protein concentration, total cell count, macrophage count, and neutrophil count compared to the CON and CUR groups. The PM-induced increased total cell count in BALF was significantly recovered to basal levels in the PM+CUR group. Furthermore, images of Hema3-stained BALF cells clearly showed that the increased proportion of macrophages in the PM group was reduced in the curcumin-treated PM group. Moreover, the absolute numbers of macrophages were also decreased in all PM+CUR groups compared to those in the PM group. Thus, the oral administration of curcumin significantly decreased lung inflammation and injury in this inhaled PM mouse model. 

### 2.2. Curcumin Downregulated the Expression of IL-1β, IL-6, and TNF-α in the Lung Tissue and BALF of Mice with PM-Induced Lung Inflammation

**Figure 2:**
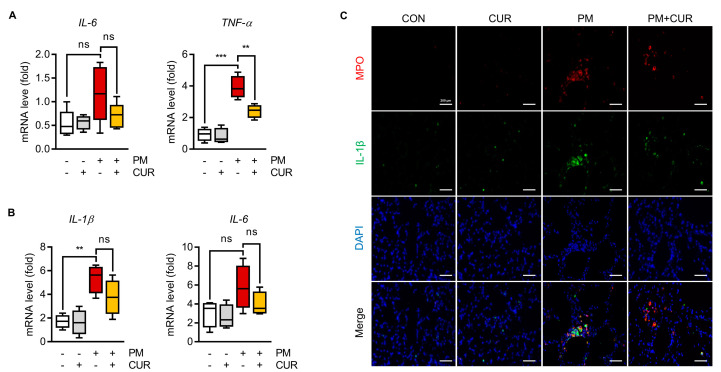
Curcumin downregulated the expression of IL-1β, IL-6, and TNF-α in PM-induced inflammation in mice lung tissue and BALF. (**A**) Effect of curcumin on PM-induced inflammatory cytokine production in lung tissue determined by RT-qPCR. (**B**) Effect of curcumin on PM-induced inflammatory cytokine production in BALF. (**C**) Co-localization of IL-1β with MPO was observed after immunofluorescence staining using indicated antibodies. Scale bars indicate 200 μm. ** *p* < 0.01, *** *p* < 0.005, and ns (not significant) indicates *p* ≥ 0.05 vs. PM-treated group. CON, control group; CUR, curcumin; PM, particulate matter (n = 6 per group).

PM-induced lung inflammation was assessed by measuring the expression levels of inflammatory cytokines, including TNF-α, IL-1β, and IL-6, in the lung tissue and BALF cells using RT-qPCR. In the lung tissue and BALF cells, the expression levels of representative cytokines, including TNF-α, IL-1β, and IL-6, were measured using RT-qPCR to evaluate pulmonary inflammation caused by PM. As expected, a significant increase in the levels of these cytokines was observed after PM treatment. PM induced the upregulation of IL-6 and TNF-α in lung tissue, while the increased levels of IL-1β and IL-6 in BALF cells were reversed by curcumin administration (Figure 2A,B). Myeloperoxidase (MPO) is a combined marker of oxidative stress and inflammation due to the activation of neutrophils and macrophages, which occur in inflammatory and infiltrative processes. Thus, we explored whether curcumin could alleviate inflammatory cell infiltration in the lung by performing immunofluorescence staining for MPO and IL-1β, a proinflammatory cytokine expressed by macrophages. As shown in Figure 2C, MPO was significantly elevated in the PM group and highly co-localized with increased IL-1β. In contrast, the curcumin-pretreated mouse group showed reduced expression levels of these molecules compared to the PM-only-treated mouse group. These experiments confirmed the anti-inflammatory effects of curcumin on PM-induced pulmonary injury in this in vivo mouse model. 

### 2.3. Curcumin Attenuated the PM-Induced NO Production and Inflammatory Cytokine Expression in RAW264.7 Macrophage Cells

**Figure 3:**
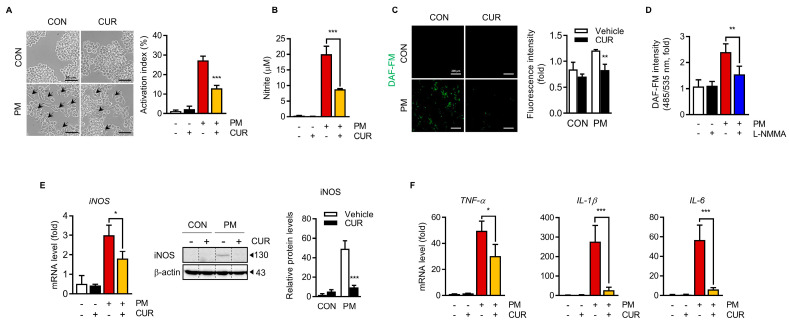
Curcumin attenuated PM-induced nitrite production and inflammatory cytokine expression in RAW264.7 macrophage cells. Effect of curcumin on (**A**) the dendritic transformation of PM-stimulated RAW264.7 cells. Cells were pre-incubated with 10 μM of curcumin for 1 h, and then treated with 20 μg/cm^2^ of PM for 24 h. Cell morphology was monitored under light microscope at ×200 magnitude. Activated RAW264.7 cells are indicated by arrows. Scale bars indicate 50 μm. The activation index percentage was expressed as the number of cells with activated morphology relative to the total number of cells, quantified as 7 random fields with total cells above 300. (**B**) Nitrite levels of RAW264.7 macrophage cells against PM-stimulated condition. RAW264.7 were pretreated with curcumin for 1 h, and incubated with PM for 12 h. Nitrite content in the cell supernatants was measured using Griess reaction assay. (**C**) Intracellular oxidative nitrosylation was monitored with fluorescence probe DAF-FM and relative intensity of fluorescence was indicated. Scale bars indicate 200 μm. (**D**) After treatment with L-NMMA, a NOS inhibitor, the levels of oxidative nitrosylation were measured by DAF-FM probe. (**E**) Expression of iNOS mRNA and protein. (**F**) Expression of proinflammatory cytokines (TNF-α, IL-1β, and IL-6) by RT-qPCR. Data presented as the mean ± SD of three independent experiments. *** *p* < 0.005, ** *p* < 0.01, * *p* < 0.05 vs. PM_10_-treated group. CON, control group; CUR, curcumin; PM, particulate matter.

Airborne PM penetrates the lungs, resulting in macrophage activation and the generation of excess oxidative stress and inflammation, which are involved in respiratory diseases. Thus, the direct effects of curcumin on PM-induced inflammatory responses were evaluated in vitro using RAW264.7 macrophage cells. Cell morphological changes after PM treatment with or without curcumin were observed using optical microscopy. The untreated control (CON) and curcumin-treated (CUR) RAW264.7 cells were circular with smooth edges devoid of pseudopodia (Figure 3A), whereas those stimulated with PM for 24 h (PM) had macrophage activation characteristics, such as an increased cell size and extended pseudopodia. However, following curcumin pretreatment for 1 h prior to 24 h treatment of PM (PM+CUR), these morphological changes in the cell structure were ameliorated. NO is a widespread signaling molecule produced in response to inflammatory stimulation by macrophages and controls various macrophage functions. Figure 3B,C show the secreted NO, indirectly measured by the nitrite concentration in the RAW264.7 cell culture supernatants, and intracellular oxidative nitrosylation levels, which can be interpreted as the relative intracellular NO level, remarkably increased by PM treatment. This increase was entirely inhibited by L-NMMA (Figure 3D), showing that the fluorescence originated from NO produced by NO synthetase. Pretreatment with curcumin for 1 h prior to PM stimulation attenuated both the secreted and intracellular NO levels. Moreover, the expression level of inducible nitric oxide synthase (iNOS), which catalyzes NO production, was increased by PM treatment. Pretreatment with curcumin prior to the PM challenge notably attenuated the enhancement in PM-induced iNOS expression (Figure 3E). TNF-α, IL-6, and IL-1β are proinflammatory cytokines that mediate inflammatory responses. RAW264.7 macrophages release cytokines in response to PM stimulation. Indeed, PM stimulation notably increased the mRNA levels of TNF-α (50-fold), IL-6 (58-fold), and IL-1β (280-fold) compared to the untreated control. However, curcumin suppressed the expression of these PM-stimulated cytokines (Figure 3F). These results indicate that curcumin effectively inhibited NO production by reducing iNOS expression and the production of proinflammatory cytokines in PM-stimulated RAW264.7 macrophages. 

### 2.4. The Inhibitory Effect of Curcumin on PM-Induced Cytokine Production Was Dependent on the Mitogen-Activated Protein Kinase (MAPK) and NF-kB Signaling Pathways

**Figure 4:**
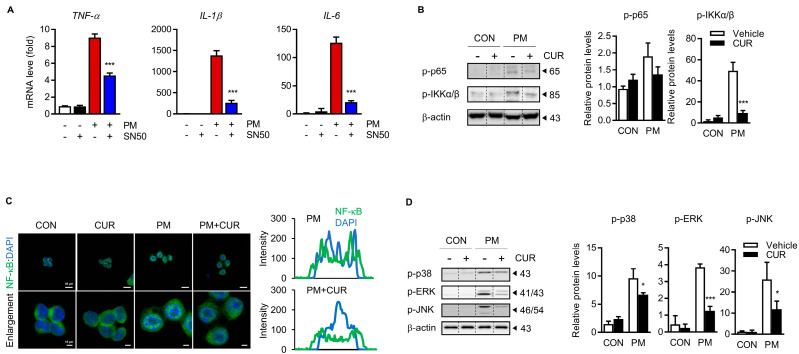
Inhibitory effect of curcumin on PM-induced cytokine production is dependent on MAPK and NF-κB signaling pathways. (**A**) NF-κB cell permeable inhibitory peptide (SN50) inhibits translocation of NF-κB and production of inflammatory cytokines. RAW264.7 cells were pretreated for 1 h with 10 μM of SN50, an inhibitor of NF-κB, and then treated for 6 h with 20 μg/mL of PM. mRNA levels of TNF-α, IL-1β, and IL-6 were assessed using RT-qPCR. (**B**) Effect of curcumin on PM-induced phosphorylation of NF-κB and IKKα/β in RAW264.7 macrophages. Activation of NF-κB was indicated by measuring the level of phosphorylation of p65 and IKKα/β via immunoblotting techniques and subsequent quantification of them with the use of ImageJ software. (**C**) Effect of curcumin on PM-induced nuclear translocation of NF-κB p65 in RAW264.7 macrophages. Confocal imaging shows subcellular distribution of NF-κB. Images show low- (**upper**; scale bars, 40 μm) and high-power magnification (**lower**; scale bars, 10 μm). Spatial profiles of fluorescence intensity of NF-κB along with DAPI, indicating nuclear DNA. (**D**) Effect of curcumin on PM-induced phosphorylation of MAPKs, including p38, ERK, and JNK, in RAW264.7 macrophages. Data represented as the mean ± SD of three independent experiments. *** *p* < 0.001, * *p* < 0.05 vs. PM-treated group. CON, control group; CUR, curcumin; PM, particulate matter.

NF-κB, the master regulator of inflammation, was targeted to explore the possible molecular mechanism of the inhibitory effect of curcumin on PM-induced proinflammatory responses. To confirm that NF-κB is involved in the effects of curcumin on RAW 264.7 cells, the NF-κB inhibitor SN50 was used to decrease NF-κB activity. Figure 4A shows that the increase in the production of TNF-α, IL-1β, and IL-6 in PM-treated cells was suppressed by SN50 administration. Activation of the NF-κB component RelA/p65 requires post-translational modification; therefore, we examined the levels of phosphorylated p65 to verify whether NF-κB was activated by PM. The results indicated that phosphorylated p65 was increased by PM treatment, whereas curcumin pretreatment blocked the PM-induced phosphorylation of p65. The phosphorylation of Ikappa kinase (IKK)α/β, an upstream regulator of NF-κB activation, was obviously enhanced by PM treatment. This increase in IKKα/β phosphorylation, however, was reversed by curcumin pretreatment. The PM-induced translocation of NF-κB from the cytoplasm to the nucleus was detected using immunocytochemistry (ICC) with an anti-p65 antibody. Overlaying the p65 (green fluorescence) and DAPI (blue fluorescence) signals in PM-treated cells showed the nuclear translocation of NF-κB, which was inhibited by curcumin. Spatial profiles of the fluorescence intensity, along with the nuclear DNA signals, confirmed the obvious nuclear translocation of NF-κB after PM stimulation and its partial inhibition by curcumin pretreatment. The MAPK pathway plays an important role in mediating the expression of proinflammatory mediators by regulating NF-κB, a major transcription factor. Therefore, we examined whether PM activated extracellular signal-regulated kinase (ERK), c-Jun N-terminal kinase (JNK), and p38 MAPK, and whether curcumin reversed this activation, using phospho-specific antibodies against MAPK proteins. Figure 4D shows that PM stimulation induced the activation of JNK, ERK, and p38, which demonstrated the regulation of inflammatory responses, and that curcumin strongly suppressed the phosphorylation of all three MAPKs. These results indicate the inhibitory effect of curcumin on PM-induced inflammation via the suppression of NF-κB by inhibiting the ERK, p38, and JNK pathways. 

### 2.5. Curcumin Reduced the PM-Induced Oxidative Stress through the Nrf2/HO-1 Pathway with Inhibition of Keap1

**Figure 5:**
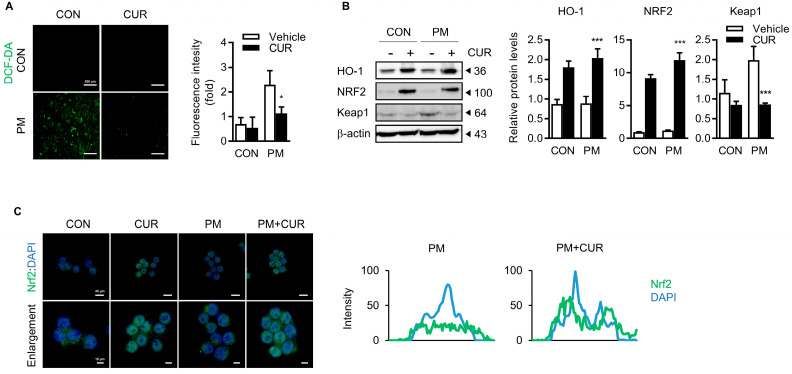
Curcumin reduced the PM-induced oxidative stress through the Nrf2/HO-1 pathway. (**A**) Intracellular ROS measurement. RAW264.7 cells were stained for 30 min using DCFH-DA (10 μM) after pretreatment of curcumin for 1 h, followed by PM treatment for 24 h. Relative fluorescence intensity was indicated. (**B**) Effect of curcumin on the PM-stimulated oxidative stress pathway-related in RAW 264.7 cells. Immunoblot analysis of Nrf2, HO-1, and Keap1 in RAW264.7 macrophages. β-actin was used as the loading control. (**C**) Effect of curcumin on nuclear translocation of Nrf2 in PM-treated RAW264.7 cells. Confocal imaging shows subcellular distribution of Nrf2. Images show low- (**upper**; scale bars, 40 μm) and high-power magnification (**lower**; scale bars, 10 μm). Spatial profiles of fluorescence intensity of Nrf2 along with DAPI, indicating nuclear DNA. Data presented as the mean ± SD of three independent experiments. * *p* < 0.05, *** *p* < 0.005 vs. PM-treated group. CON, control group; CUR, curcumin; PM, particulate matter.

Inhaled PM results in the increased production of ROS, a signaling molecule, and oxidative stress, triggering molecular events for the downstream induction of inflammation and tissue injury. Thus, we investigated whether curcumin affected the PM-induced intracellular ROS production in RAW264.7 macrophages. As expected, PM-stimulated RAW264.7 cells showed an increase of over three-fold in the intracellular ROS levels than the untreated control cells, which was significantly attenuated by curcumin pretreatment (Figure 5A). Under oxidative stress conditions, Nrf2 plays an important role in modulating antioxidant activity as a master transcription factor. To control redox homeostasis, Nrf2 dissociates from Kelch-like ECH-associated protein 1 (Keap1), a chief negative regulator of Nrf2, and translocates into the nucleus from the cytoplasm and binds to ARE, leading to the transcription of antioxidant genes. Immunoblot analyses were performed to compare the expression of Keap1, Nrf2, and its downstream target gene, HO-1. The results shown in Figure 5B demonstrate that the protein expression levels of HO-1 and Nrf2 were significantly enhanced with the treatment of curcumin. While the expression of Nrf2 and HO-1 was not affected by PM treatment, the expression of the Keap1 protein was markedly higher with PM treatment than in the untreated control (CON and CUR). This increased expression of Keap1 was significantly downregulated by the pretreatment of curcumin. As shown in the immunofluorescence images in Figure 5C, curcumin treatment clearly promoted the nuclear translocation of Nrf2, as confirmed by the spatial profiles of fluorescence intensity, along with the nuclear DNA. This suggested that PM-induced oxidative stress was prevented by curcumin pretreatment via the inhibition of the Keap1 protein and consequent activation of Nrf2, which resulted in a significant increase in the antioxidant molecule HO-1. 

## 3. Discussion

PM is an important environmental pollutant that promotes respiratory diseases by inducing an inflammatory response [14,16,18]. The inhalation of PM induces an immune response initiated by alveolar macrophages and airway epithelial cells in the lungs. Macrophages plays a key role in local inflammation due to being several times more potent in producing proinflammatory mediators, which contribute to the subsequent systemic inflammatory response [29,30]. Curcumin, a natural anti-inflammatory drug, is safe and well-tolerated at high concentrations, without inducing toxicity [31,32]. These therapeutic effects of curcumin have potential applications in various disorders, including pulmonary diseases [23,33]. Recent studies have investigated the beneficial effects of curcumin on air-pollution-induced pulmonary injury and the underlying molecular mechanisms in several types of cells. According to these findings, curcumin alleviates airway inflammation by suppressing NF-kB activation in bronchial epithelial cells and reduces ROS production by activating Nrf2 pathways in nasal fibroblast cells [27,28,34,35].

In the current study, an in vivo mouse model of PM-induced pulmonary inflammation was established to verify the protective effects of curcumin against PM exposure-induced damage. Intratracheal instillation was selected as the administration method to deliver the PM to the trachea in a quick, accurate manner. Two days after instillation, pulmonary inflammation was assessed by lung histopathology. After inhalation, PM was deposited in the lungs, mainly in the alveoli (Figure 1A). The most important pulmonary inflammation signs observed in this study included a damaged alveolar structure and inflammatory cell filtration, which were significantly enhanced in lung tissue exposed to PM. BALF, commonly used in the evaluation of lung disease, also revealed a pulmonary inflammatory response, with increased total protein levels and inflammatory cells, such as macrophages, in BALF. Curcumin-pretreated mouse lungs were significantly protected from PM-induced inflammation and injury, as demonstrated by histological analysis, with a comparable number of inflammatory cells in BALF. Similarly, the PM-induced proinflammatory cytokines, whose overproduction leads to cell death and tissue damage, were remarkably attenuated by curcumin treatment.

Macrophages have been implicated in a wide spectrum of disorders, including pulmonary diseases [36,37]. Air pollutants, including PM, induce an immune response initiated by alveolar macrophages and amplified by bloodborne infiltrated macrophages [13,38,39]. The murine macrophage RAW264.7 cell line is widely used as a model to mimic inflammation to evaluate the potential preventive effects of supplements and drugs. In this study, we used the RAW264.7 cell line to verify the PM-induced inflammatory response and evaluate the effect of curcumin on the PM-induced inflammatory response in vitro. We clearly observed the characteristics of the inflammatory activation of RAW264.7 cells after PM stimulation, such as morphological changes, elevated NO production with enhanced iNOS expression levels, and increased levels of proinflammatory cytokines, including TNF-a, IL-1β, and IL-6. These effects were significantly attenuated by pretreatment with curcumin, indicating the preventive capacity of curcumin against PM-stimulated macrophage activation.

The activation of macrophages is initiated by a series of cellular responses, including activation of the MAPK cascade (including ERK, p38, and JNK) and NF-κB signaling to amplify the production of inflammatory cytokines [40,41,42,43]. Using a selective blocker of NF-κB (SN50), we verified that PM-induced cytokine production occurred mainly through NF-κB (Figure 4A) signaling. Furthermore, we verified that curcumin suppressed the PM-induced activation of NF-κB, indicated by its phosphorylation and nuclear translocation, by diminishing MAPK signaling, especially for ERK and JNK. While NF-κB plays a major role in the inflammatory response, the Nrf2 signaling pathway is critical for cytoprotection against oxidative stress [44]. The activated Nrf2 translocates to the nucleus to transcribe several proteins that are involved in cellular defenses, such as antioxidant enzymes [45]. Since interference exists between these two pathways [46], the inhibition of NF-κB and induction of Nrf2 are considered the best approaches to prevent PM-induced inflammatory diseases. Curcumin also appeared to significantly attenuate the PM-induced production of ROS in macrophages by activating the Nrf2/HO-1 axis with the inhibition of Keap1 (Figure 5). Thus, our results provide sufficient evidence that curcumin is a dietary supplement that can potentially prevent pulmonary disease because of its bimodal effect—namely, the suppression of NF-κB and induction of Nrf2—against PM-induced inflammation.

In summary, we demonstrated the protective effect of curcumin against PM-induced pulmonary inflammation in a well-established in vivo mouse model. Furthermore, we provided evidence that curcumin suppressed the PM-induced inflammatory activation of macrophages, especially the production of proinflammatory mediators, through the regulation of MAPK/NF-κB signaling. Curcumin also appeared to attenuate ROS generation by the Nrf2/HO axis. These results suggest that curcumin prevents oxidative stress and pulmonary inflammation induced by air pollution.

## 4. Materials and Methods

### 4.1. Animals and Reagents

All animal procedures were approved by the Institutional Animal Care and Use Committee (IACUC) of Korea University (Seoul, Republic of Korea; Approval No: KUIACUC-2021-0078). Eight-week-old C57BL/6N male mice were purchased from RaonBio, Inc. (Gyeonggi-do, Republic of Korea). Mice were housed in cages at room temperature and maintained at 23 ± 1 °C at a relative humidity of 55 ± 5% with a 12 h light/dark cycle. The animals were allowed at least one week to adapt to their laboratory housing environment, and food and water were available ad libitum. ERM-CZ100 fine dust (similar to PM10) (European Commission Joint Research Centre, Geel, Belgium) was purchased through Sigma. This substance contained approximately 0.5 g of fine dust and was processed similarly to PM10, and its main components were PAHs, including benzo[*a*]anthracene, indeno [1,2,3-*c,d*]pyrene, and benzo[*b*]fluoranthene. Curcumin powder, polyethylene glycol 400, and NF-κB SN50 were purchased from Sigma-Aldrich (St. Louis, MO, USA). N-methyl-L-arginine (L-NMMA) was purchased from Thermo Fisher Scientific (Waltham, MA, USA).

### 4.2. PM Inhalation and Curcumin Treatment

Mice were randomly divided into four groups (n = 6 per group). The CON and PM groups received 50% polyethylene glycol in phosphate-buffered saline (PBS), and the CUR group and PM+CUR group received curcumin dissolved in the same solution by oral administration once daily at a dose of 50 mg/kg for seven consecutive days prior to PM exposure. The PM in sterile saline was mixed in certain concentrations and was sonicated for 5 min before use. PM (10 mg/kg, saline for the CON and CUR groups) was administered using the nonsurgical intratracheal instillation method [47]. Briefly, the mouse was placed on an angled platform hanging by its incisors on the wire and gently laid down; the mouse was placed under anesthesia with isoflurane. Fifty microliters of saline, as described above, was dropped into the trachea using a sterile syringe and laryngoscope. Two days after PM exposure, all animals were euthanized with an overdose of 3–5% isoflurane. BALF and lung tissues were harvested for subsequent experiments.

### 4.3. Collection and Analysis of BALF

Tracheostomy was performed on freshly obtained lung tissues from sacrificed mice [48]. First, a plastic tube was intubated into the trachea, and 1 mL of precooled and pyrogen-free PBS was instilled into the lung through the tracheal cannula using a sterile syringe. The BALF was collected after the lungs were gently massaged for some time, and this collection process was repeated thrice. The collected BALF was centrifuged at 500× *g* for 5 min at 4 °C. The total protein levels in the BALF supernatant were measured using a BCA protein assay kit. The cell pellet was resuspended in 200 μL of saline and used for total cell counts with an EVE^TM^ automatic cell counter (NanoEnTeK, Seoul, Republic of Korea). Then, to count macrophages and neutrophils in BALF, 5 × 10^2^ cells were counted per animal, seeded on glass slides, and stained with hema3 (Fisher Scientific, Middlesex County, MA, USA).

### 4.4. Lung Histopathology and Immunofluorescence Analysis

To standardize the fixation procedures for histological quantification, tracheostomy and intubation fixation were combined with the general tissue fixation method. Briefly, after the trachea was incised, airway intubation was performed using a flexible catheter, and 4% paraformaldehyde was injected into the lungs at a pressure equivalent to 25 cm of the liquid. Mouse lung tissues were post-fixed in 4% paraformaldehyde overnight, embedded in paraffin, and cut into 4-μm-thick histological sections using a microtome (Leica, Wetzlar, Germany). After deparaffinization, lung sections were rehydrated by immersing them in a diluted series of ethanol solutions. For histopathological analysis, rehydrated lung sections were stained with H&E (Sigma), and images were acquired using a Leica ICC50 E microscope (Leica). Antigen retrieval was performed by heating the slides in a sodium citrate buffer for 10 min in a steamer, followed by cooling for 20 min and rinsing in PBS. The sections were then incubated in 0.5% Triton X-100 in PBS for 10 min for permeabilization, followed by blocking with 3% BSA in PBS with 0.3% Triton X-100 for 1 h at room temperature. Incubation of tissue sections with indicated primary antibodies (anti-IL1β; 1:100, Cell Signaling Technology, Danvers, MA, USA; catalog # 83186T, anti-MPO; 1:100, R&D Systems, Minneapolis, MI, USA; catalog # AF3667) was conducted overnight at 4 °C and then with fluorophore-conjugated secondary antibodies (Alexa Fluor 488 and 594 1:1000; Invitrogen, Waltham, MA, USA) for 1 h at room temperature. Images were analyzed using a Zeiss Axiovert 200 inverted microscope (Carl Zeiss AG, Oberkochen, Germany).

### 4.5. RNA Extraction and qPCR Analysis

RNA was extracted from three samples: lung tissue, immune cells in BALF, and RAW264.7 cells. Lung tissues were collected and stored directly in RNAlater Stabilization Solution (Invitrogen). Total RNA was extracted using TRIzol reagent (Invitrogen), and the concentration and quality of the extracted RNA were determined using a spectrophotometer. RNA with an absorption ratio (OD_260_ nm/OD_280_ nm) of approximately 1.8 was selected and converted into cDNA using the Reverse Transcriptase Premix for qPCR (Biofact, Daejeon, Republic of Korea). GoTaq® qPCR Master (Promega, Madison, WI, USA) was used for PCR amplification on the ABI 7500 Real-Time PCR System (Applied Biosystems, Middlesex County, MA, USA). To isolate immune cells from the total cells in BALF, the collected BALF was mixed with high-glucose Dulbecco’s Modified Eagle’s Medium (DMEM) (Invitrogen, Waltham, MA, USA) supplemented with 10% heat-inactivated fetal bovine serum (Thermo Fisher Scientific, Waltham, MA, USA) and seeded on a poly-l-lysine-coated 24-well plate at 37 °C in a humidified 5% CO_2_ and 95% air atmosphere for 3 h. The supernatant was removed when the immune cells were attached, and RNA extraction was carried out in the same manner as for lung tissue and RAW264.7 cells. Gene expression levels were normalized to those of GAPDH. The primers used in our study were as follows: GAPDH (Forward 5′-ACCACATGCCATCAC-3′, Reverse 5′-CACCACCCTGTTGCC-3′), TNF-α (Forward 5′-GGTGCCTATGTCTCAGCCTCTT-3′, Reverse 5′-GCCATAGAACTGATGAGAGGGAG-3′), IL-6 (Forward 5′-CTCTGCAAGAGACTTCCATCCA-3′, Reverse 5′-TTGTGAAGTAGGGAAGGCCG-3′), IL-1β (Forward 5′-TGCCACCTTTTGACAGTGATG-3′, Reverse 5′-TTGGAAGCAGCCCTTCATCTT-3′), iNOS (Forward 5′-GAGACAGGGAAGTCGAAGCAC-3′, Reverse 5′-CCAGCAGTAGTTGCTCCTCTTC-3′).

### 4.6. Cell Culture

The murine macrophage cell line RAW 264.7, purchased from the Korea Cell Line Bank (Seoul, Republic of Korea), was cultured in high-glucose DMEM (Invitrogen) supplemented with 10% fetal bovine serum (Invitrogen) and 1% penicillin–streptomycin at 37 °C in a humidified 5% CO_2_ incubator. When the confluence of these cells reached approximately 80%, the cells were carefully detached by pipetting the refreshed medium. Then, cells were seeded in a 60-mm dish, and the same procedure was repeated after three days to maintain the cells. Cell passages were performed up to 10 times from the initial passage.

### 4.7. Nitrite Assay

RAW264.7 macrophages were plated at a density of 1 × 10^4^ cells/well in 96-well plates and pretreated with curcumin (10 μM) for 1 h and then incubated with PM (20 μg/cm^2^) for 24 h. Nitrite levels were determined using the Griess reagent. Briefly, a dilution series of nitrite used as the standard reference curve and 50 μL of each cell cultured medium were transferred to the wells of the 96-well microplate, and 50 μL of the Griess solution was added to each well. The samples were then incubated for 5–10 min at 37 °C in the dark. The fluorescence intensity was then measured using a Multiskan SkyHigh Microplate Spectrophotometer (Thermo Fisher Scientific) at 540 nm.

### 4.8. Intracellular Detection of Oxidative Nitrosylation

Intracellular levels of oxidative nitrosylation were detected to indirectly assess intracellular NO. We plated 1.5 × 10^5^ cells/well in 24-well plates without phenol red to avoid fluorescence interference. After overnight treatment with curcumin and PM, the cells were incubated with 5 μM of DAF-FM diacetate at 37 °C with 5% CO_2_ for 1 h. After incubation, the cells were maintained for an additional 30 min in fresh medium to allow complete de-esterification of the intracellular diacetate. Then, the cells were then washed with PBS, and fluorescence was measured using a Perkin Elmer 1420 multilabel counter (PerkinElmer, Waltham, MA, USA) at the excitation and emission wavelengths of 485 nm and 535 nm, respectively. The values were normalized to the protein concentrations of each sample using a BCA protein assay kit (Thermo Fisher Scientific). The specificity of DAF-FM, as an NO probe, was checked by treating the cells with 1 mM L-NMMA (a cell-permeable inhibitor of NOS, Sigma) for 24 h.

### 4.9. Intracellular ROS Assessment

Intracellular ROS levels were detected using 6-carboxy-2′,7′-dichlorodihydrofluorescein diacetate (H2DCF-DA) (Invitrogen). RAW264.7 cells were seeded at a density of 1.5 × 10^5^ cells/well in 24-well plates and treated with curcumin and PM as described above. After overnight incubation with an additional 10 μM H2DCF-DA, the cells were incubated at 37 °C for 30 min. The cells were then washed with PBS, and fluorescence was measured using a Perkin Elmer 1420 multilabel counter (PerkinElmer, Waltham, MA, USA) at the excitation and emission wavelengths of 485 nm and 535 nm, respectively. The values were normalized to samples’ protein concentrations using a BCA protein assay kit (Thermo Fisher Scientific) [49]. The samples were tested in triplicate in three individual experiments.

### 4.10. Immunoblot Analysis

To detect protein expression levels, cells were lysed in RIPA lysis buffer (Thermo Fisher Scientific) containing protease inhibitors. Protein concentrations were determined using a BCA protein assay kit (Thermo Fisher Scientific, catalog # 23225) according to the manufacturer’s protocol. Total protein extracts were separated using sodium dodecyl sulfate–polyacrylamide gel electrophoresis (8–15% depending on protein molecular weights), and proteins were transferred onto polyvinylidene difluoride membranes (Bio-Rad, Hercules, CA, USA; catalog # 1620264), followed by membrane blocking with 3% bovine serum albumin in Tris-buffered saline with 0.05% Tween-20. After probing with the appropriate primary antibodies diluted 1:1000 in the blocking solution (anti-iNOS; Cell Signaling Technology, catalog # 13120, anti-phospho-p65; Cell Signaling Technology, catalog # 3033, anti-phospho-IKKα/β; Cell Signaling Technology, catalog # 2697S, anti-phospho-p38; Cell Signaling Technology, catalog # 9215, anti-phospho-ERK; Abcam, catalog # ab201015, anti-phospho-JNK; Santa Cruz, catalog # sc6254, anti-HO-1; Abcam, catalog # ab13243, anti-Nrf2; Cell Signaling Technology, catalog # 12721S, anti-Keap1; Cell Signaling Technology, catalog # 8047S, anti-β-actin; Invitrogen, catalog # MAI-140), protein expression levels were visualized using horseradish peroxidase-labeled secondary antibodies and an enhanced chemiluminescence detection kit (Bio-Rad). Antibody-specific bands were detected using a chemiluminometer (ImageQuant^TM^ LAS4000; GE Healthcare, Chicago, IL, USA). Protein bands were quantified using the ImageJ software.

### 4.11. Quantification and Statistical Analysis

Data are expressed as the mean ± standard error of the mean (SEM), and experiments were performed at least thrice. *T*-test and one-way ANOVA were used to determine the statistical significance between two or more than three mean values. GraphPad Prism 7 (GraphPad, San Diego, CA, USA) was used for statistical analysis. A *p* value of <0.05 was considered statistically significant.

## Data Availability

The authors acknowledge that the data presented in this study must be deposited and made publicly available in an acceptable repository.
